# Association of oXiris Hemoadsorption with Inflammatory, Hemodynamic, Respiratory, and Organ Dysfunction Parameters in Patients with Sepsis and Septic Shock in the Intensive Care Unit: A Retrospective Comparative Cohort Study

**DOI:** 10.3390/life16081367

**Published:** 2026-08-19

**Authors:** Semiha Orhan, Murat Ay, Merve Ay, Kemal Yetis Gulsoy

**Affiliations:** 1 Department of Internal Medicine, Intensive Care Unit, Faculty of Medicine, Afyonkarahisar Health Sciences University, Afyonkarahisar 03030, Türkiye; 2 Kütahya State Hospital, Kutahya 43100, Türkiye; muratay09@gmail.com; 3 Kütahya Evliya Celebi Training and Research Hospital, Kutahya 43040, Türkiye; mrvkcsln.dr@gmail.com; 4 Burdur Public Hospital, Burdur 15100, Türkiye; kemalgulsoy@gmail.com

**Keywords:** sepsis, septic shock, oXiris, hemoadsorption, extracorporeal blood purification, cytokine adsorption, organ dysfunction, sequential organ failure assessment score, intensive care unit, endotoxin adsorption, propensity score

## Abstract

Background/Objectives: This study aimed to evaluate the associations of adding oXiris hemoadsorption therapy to standard sepsis treatment with inflammatory markers, organ dysfunction, hemodynamic parameters, respiratory function, and overall survival (OS) in patients with sepsis and septic shock managed in the intensive care unit (ICU). In our ICU, oXiris was used as adjunctive hemoadsorption for endotoxin and cytokine removal, delivered on a continuous venovenous hemodiafiltration (CVVHDF) platform and initiated independently of acute kidney injury (AKI). Methods: This retrospective comparative cohort study included 83 adult patients with sepsis or septic shock admitted to the Internal Medicine ICU. Patients were allocated to either the oXiris group (*n* = 33), which received oXiris hemoadsorption for 72 h in addition to standard sepsis therapy, or the control group (*n* = 50), which received standard sepsis therapy without oXiris or another adsorptive CRRT membrane. Conventional intermittent hemodialysis for independent renal indications was permitted and recorded in both groups. C-reactive protein (CRP), procalcitonin (PCT), white blood cell count, updated Sequential Organ Failure Assessment (SOFA-2) score, PaO_2_/FiO_2_ ratio, serum lactate, norepinephrine dose, and renal parameters were recorded at baseline, 48 h, and 72 h. Between-group differences in temporal changes were evaluated using adjusted generalized estimating equation models, and robustness was assessed with a propensity-score-based inverse-probability-of-treatment-weighted (IPTW) sensitivity analysis. Survival analysis was performed using the Kaplan–Meier method and log-rank test. Results: The oXiris group had significantly greater baseline clinical severity, including a higher burden of renal dysfunction. This marked baseline imbalance was considered important when interpreting the unadjusted findings and supported the use of adjusted and IPTW analyses. In the oXiris group, CRP, PCT, SOFA-2 score, and norepinephrine requirement showed greater reductions over time, whereas the PaO_2_/FiO_2_ ratio improved significantly. After IPTW adjustment, the between-group differences in SOFA-2 score, norepinephrine requirement, and PaO_2_/FiO_2_ ratio remained statistically significant, whereas the CRP and PCT differences were no longer statistically significant. Serum lactate levels showed an increasing trend in the oXiris group compared with the control group (*p* = 0.042). No significant difference in overall survival was observed between the two groups (*p* = 0.897). Conclusions: The addition of oXiris hemoadsorption to standard sepsis therapy was associated with favorable early temporal changes in organ dysfunction, norepinephrine requirement, and oxygenation. However, the observed CRP and PCT differences were not preserved after IPTW adjustment, and no survival association was identified. These findings should therefore be interpreted as associations rather than evidence of a causal treatment effect.

## 1. Introduction

Sepsis is a life-threatening acute organ dysfunction syndrome caused by a dysregulated host response to infection [1,2]. Its pathophysiology is driven by uncontrolled inflammatory response, endothelial dysfunction, microcirculatory impairment, coagulation activation, and metabolic dysregulation. The progression of these processes may result in the development of disseminated intravascular coagulation, tissue hypoxia, multiple organ dysfunction syndrome, and death [1,2].

Sepsis remains a major public health burden worldwide, with an estimated 48.9 million new sepsis cases and 11 million sepsis-related deaths annually [3]. Sepsis-related mortality in the intensive care unit (ICU) remains high, particularly in patients with septic shock, where cellular, metabolic, and circulatory dysfunction is most pronounced [4,5,6]. Furthermore, sepsis imposes a substantial burden on healthcare systems through prolonged ICU stays, mechanical ventilation requirements, long-term functional decline, and high healthcare costs [4].

According to the Surviving Sepsis Campaign 2021 guidelines, early broad-spectrum antimicrobial therapy, appropriate fluid resuscitation, early vasopressor support, source control, and organ support strategies are the cornerstone of sepsis management [6]. However, failure to control the inflammatory response despite standard therapy may lead to organ dysfunction progression and persistent mortality risk. Therefore, extracorporeal blood purification strategies targeting the removal of endotoxins, proinflammatory cytokines, and other inflammatory mediators have gained attention as adjuvant approaches in sepsis treatment [7,8].

The oXiris filter is a modified acrylonitrile-co-methallyl sulfonate surface-treated (AN69ST)-based membrane surface-coated with positively charged polyethyleneimine and heparinized to provide local antithrombotic activity. This design enables oXiris to integrate endotoxin adsorption, cytokine adsorption, continuous renal replacement therapy (CRRT), and local anticoagulation within a single system [9]. Clinical and experimental data indicate that the oXiris membrane may reduce the burden of inflammatory mediators, may contribute to hemodynamic stabilization, and has been associated with improvements in organ dysfunction parameters [10,11].

However, the available clinical evidence remains inconsistent. Randomized studies have generally been small and have primarily demonstrated effects on endotoxin levels, inflammatory mediators, and short-term physiological parameters rather than a definitive survival benefit [11,12,13]. Similarly, systematic reviews and meta-analyses have reported potentially favorable effects on organ dysfunction and short-term mortality, but their conclusions are limited by substantial heterogeneity, small sample sizes, and the predominance of observational studies [14,15]. Accordingly, current international sepsis guidelines do not recommend the routine use of extracorporeal blood purification therapies in unselected patients with sepsis or septic shock because of insufficient high-certainty evidence [6].

Several studies have compared the oXiris membrane with other extracorporeal filter systems or conventional CRRT membranes. However, few studies have directly compared oXiris hemoadsorption combined with standard sepsis therapy alone. Therefore, this study aimed to examine the associations of adding oXiris hemoadsorption to standard sepsis therapy, compared with standard therapy alone, with early inflammatory, hemodynamic, respiratory, and organ dysfunction parameters and overall survival (OS) in patients with sepsis or septic shock in the ICU.

## 2. Materials and Methods

### 2.1. Study Design and Ethical Approval

This retrospective comparative cohort study was conducted in the Internal Medicine ICU of Afyonkarahisar Health Sciences University Faculty of Medicine between January 2022 and December 2025. This study was approved by the Clinical Research Ethics Committee of Afyonkarahisar Health Sciences University (decision number: 2006/7; approval date: 5 June 2026) and conducted in accordance with the principles of the Declaration of Helsinki.

### 2.2. Patient Selection and Study Groups

This study included patients with a diagnosis of sepsis or septic shock who were managed in the ICU during the study period. Sepsis and septic shock were diagnosed based on the Third International Consensus Definitions (Sepsis-3) criteria using the Sequential Organ Failure Assessment (SOFA) score [1]. Organ dysfunction was assessed using the updated SOFA-2 scoring system, which was developed and validated in contemporary ICU populations [16,17]. Patients were categorized into two groups: the oXiris group, which received oXiris hemoadsorption in addition to standard sepsis therapy, and the control group, which received only routine standard sepsis therapy. All patients received standard sepsis management in accordance with the Surviving Sepsis Campaign guidelines [6]. Standard treatment included fluid resuscitation, empirical or culture-directed antimicrobial therapy, source control, vasoactive agent support, and organ support therapies.

Indication for oXiris hemoadsorption. In our ICU, oXiris was used as an adjunctive extracorporeal blood purification strategy in patients with sepsis or septic shock who exhibited a hyperinflammatory phenotype with progressive or vasopressor-dependent circulatory failure despite guideline-based standard therapy. The primary therapeutic intent of oXiris was the adsorption of circulating endotoxin and proinflammatory mediators rather than renal replacement per se; the CVVHDF circuit served only as the delivery platform for the adsorptive membrane. oXiris was initiated for hemoadsorptive rather than renal-replacement intent. Nevertheless, because it was delivered in CVVHDF mode, the adsorptive effects of the membrane cannot be fully separated from concomitant diffusive and convective clearance and fluid management. The decision to initiate oXiris therapy was made by the treating intensivist based on clinical judgment according to routine clinical practice, rather than predefined objective study allocation criteria. The decision was based on clinical severity (high or escalating norepinephrine requirement, hyperlactatemia, a rising organ dysfunction score, and laboratory markers of hyperinflammation), and oXiris initiation was not contingent on a renal indication, a documented AKI diagnosis, or Kidney Disease: Improving Global Outcomes (KDIGO) stage. Consequently, oXiris was not restricted to patients with a renal indication, and most patients received it primarily for immunomodulation, with renal support—when present—being a secondary effect.

The control group comprised patients managed with guideline-based standard therapy who did not receive oXiris or any other adsorptive. Conventional intermittent hemodialysis for a renal indication (fluid overload, refractory electrolyte or acid-base disturbance, or uremia) was permitted in both groups when clinically required and was recorded as a concomitant treatment variable; no control patient received oXiris at any time. The comparison was therefore between oXiris hemoadsorption added to standard therapy and standard therapy without hemoadsorption, not between two different purification membranes.

### 2.3. Eligibility Criteria

The inclusion criteria were (1) age ≥ 18 years, (2) diagnosis of sepsis or septic shock in the ICU according to the Sepsis-3 criteria, (3) receipt of uninterrupted oXiris hemoadsorption therapy for 72 h in addition to standard sepsis treatment (the oXiris group), (4) receipt of standard sepsis therapy with at least 72 h of follow-up (the control group), and (5) availability of complete records for all clinical and laboratory parameters.

The exclusion criteria were (1) age < 18 years, (2) unconfirmed sepsis diagnosis (clinical or laboratory), (3) total length of ICU stay < 72 h, (4) discontinuation of oXiris hemoadsorption before 72 h due to clinical, technical, or mortality-related reasons, (5) terminal malignancy, and (6) incomplete clinical follow-up or laboratory data in patient records.

### 2.4. Extracorporeal Treatment Protocol

Vascular access was established in the oXiris group through the femoral or internal jugular vein using a double-lumen catheter (11F–13.5F). All sessions were delivered in continuous venovenous hemodiafiltration (CVVHDF) mode using the Prismaflex CRRT system (Baxter, Deerfield, IL, USA) with the oXiris (Baxter) adsorptive membrane set, with standardized parameters of a blood flow rate of 120–150 mL/min and a total treatment dose of 30 mL/kg/h. Prism0cal B22, Prism0cal, and Dialisan (Baxter) were used as CRRT dialysis solutions during oXiris-CVVHDF. All three formulations contained 3 mmol/L lactate after reconstitution. Anticoagulation was preferentially performed with heparin or regional citrate anticoagulation, selected based on each patient’s clinical contraindications. Filter sets were exchanged every 72 h in accordance with the manufacturer’s specified maximum safe service life to prevent mechanical damage to pump segments [18].

The total effluent dose was delivered on the CVVHDF platform solely as the vehicle for membrane-based endotoxin and cytokine adsorption; oXiris initiation was driven by the hyperinflammatory and hemodynamic indication described above rather than by a renal indication. When a concurrent renal indication for conventional intermittent hemodialysis was present, this was provided and recorded separately.

### 2.5. Data Collection and Outcome Measures

The baseline time point (hour 0) was defined as the moment of sepsis or septic shock diagnosis in both groups. In the oXiris group, hemoadsorption was initiated simultaneously with standard sepsis therapy without delay after diagnosis. Therefore, the baseline assessment in both groups corresponded to the same clinical time point (time of diagnosis). This ensured that temporal comparisons between groups were made based on a common, symmetric reference point. Clinical and laboratory assessments were performed at baseline, 48 h, and 72 h in both groups.

C-reactive protein (CRP), procalcitonin (PCT), white blood cell count (WBC), serum lactate, PaO_2_/FiO_2_ ratio, norepinephrine dose (μg/kg/min), and Sequential Organ Failure Assessment (SOFA-2) score were recorded to assess treatment-associated temporal changes.

In addition, baseline and serial renal parameters (serum creatinine and estimated glomerular filtration rate), a history of chronic kidney disease, and the use of conventional intermittent hemodialysis for a renal indication were recorded in both groups. Because reliable pre-morbid baseline creatinine values were not uniformly available, formal Kidney Disease: Improving Global Outcomes (KDIGO) AKI staging could not be applied retrospectively; renal status is therefore reported descriptively.

The primary outcomes were temporal changes in the SOFA-2 score, norepinephrine requirement, CRP levels, PCT levels, and PaO_2_/FiO_2_ ratio. The secondary outcomes were temporal changes in serum lactate levels and overall survival.

### 2.6. Statistical Analysis

All statistical analyses were performed using IBM SPSS Statistics version 26.0 (IBM Corp., Armonk, NY, USA). Categorical variables were presented as frequencies and percentages. The normality of continuous variables was assessed using the Shapiro–Wilk test. Normally distributed continuous variables were presented as mean ± standard deviation, whereas nonnormally distributed variables were presented as median and interquartile range (IQR). Between-group comparisons were performed using the chi-square test or Fisher’s exact test for categorical variables, the independent-samples *t*-test for normally distributed continuous variables, and the Mann–Whitney U test for nonnormally distributed continuous variables. Within-group temporal changes were evaluated using the Friedman test for nonnormally distributed repeated measures. When the Friedman test was significant, pairwise comparisons were performed using the Bonferroni-corrected Wilcoxon signed-rank test.

Adjusted generalized estimating equations (GEE) models were applied to evaluate whether temporal changes in repeated clinical and laboratory parameters differed between groups. The models included group, time, and the group × time interaction term. An exchangeable working correlation matrix was used to model within-subject correlation across repeated measurements.

Age, SOFA-2 score, lactate level, norepinephrine dose, and mechanical ventilation status were considered potential covariates in adjusted analyses. Covariates were selected according to the dependent variable under analysis to prevent overadjustment and multicollinearity. Covariates for inflammatory and respiratory parameters were age, mechanical ventilation status, baseline SOFA-2 score, and lactate level, whereas covariates for hemodynamic and organ dysfunction parameters were age, mechanical ventilation status, lactate/SOFA-2 score (depending on the dependent variable), and baseline norepinephrine dose. Results were reported with Wald chi-square statistics and *p*-values. Adjusted regression coefficients (*β*) with 95% confidence intervals (CIs) for the group × time interaction terms and adjusted mean between-group differences at each time point (48 and 72 h) were calculated using linear contrasts.

To mitigate confounding by indication, a sensitivity analysis was performed using a propensity score for oXiris treatment derived by logistic regression from baseline age, SOFA-2 score, lactate, norepinephrine dose, mechanical ventilation, and serum creatinine. Stabilized inverse-probability-of-treatment weights (IPTW) were calculated, covariate balance was assessed using standardized mean differences (balance defined as an absolute value < 0.10), and the adjusted oXiris-control differences in change from baseline to 72 h were re-estimated using weighted least squares regression.

Overall survival was defined as the time from sepsis or septic shock diagnosis to death during the index hospitalization. Patients discharged alive were censored on the date of hospital discharge. Survival analysis was performed using the Kaplan–Meier method, and comparisons between groups were performed using the log-rank test. Crude in-hospital mortality proportions were compared using Fisher’s exact test. Statistical significance was set at a two-sided *p*-value < 0.05. Lactate levels were analyzed and reported in mg/dL, as available in the institutional laboratory information system.

## 3. Results

**Figure 1:**
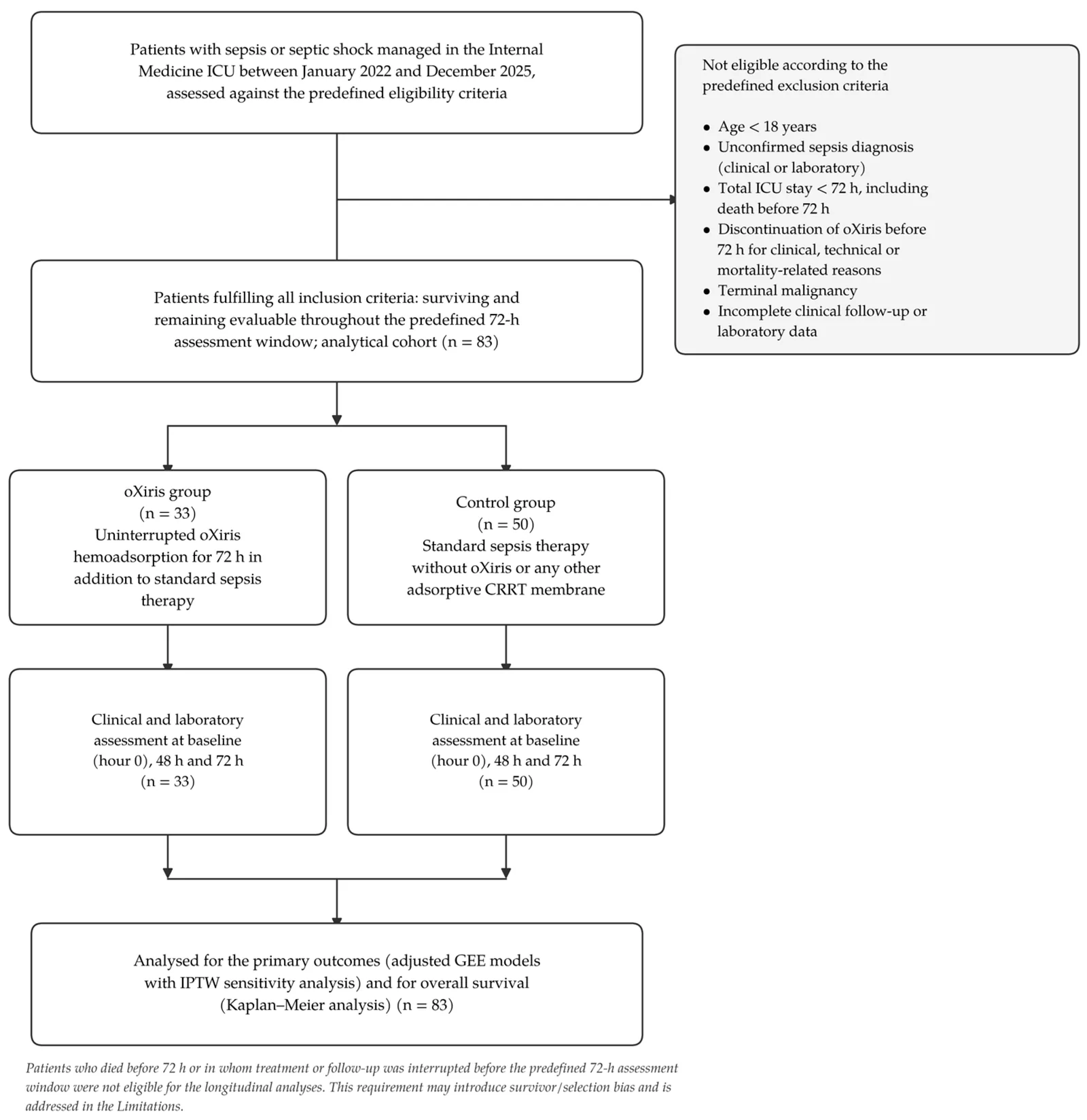
Patient flow diagram. Eligibility required uninterrupted treatment and complete follow-up throughout the predefined 72-h assessment window; patients who died before 72 h or in whom oXiris hemoadsorption was discontinued before 72 h were therefore not eligible for the longitudinal analyses. This requirement is a potential source of survivor/selection bias and is addressed in the Limitations. CRRT, continuous renal replacement therapy; GEE, generalized estimating equations; ICU, intensive care unit; IPTW, inverse-probability-of-treatment weighting.

Patients with sepsis or septic shock managed in the Internal Medicine ICU during the study period were assessed against the predefined eligibility criteria described in Section 2.3. Because inclusion required uninterrupted treatment and complete clinical and laboratory follow-up throughout the predefined 72-h assessment window, patients whose total ICU stay was shorter than 72 h, including those who died before 72 h, and patients in whom oXiris hemoadsorption was discontinued before 72 h for clinical, technical, or mortality-related reasons were not eligible and could therefore not contribute to the longitudinal analyses; this restriction is addressed as a potential source of survivor/selection bias in the Limitations. A total of 83 patients fulfilled all eligibility criteria and constituted the analytical cohort. The patient flow is summarized in Figure 1.

### 3.1. Demographic Characteristics and Baseline Clinical Features

A total of 83 patients were included in this study. Of the 83 patients, 33 were included in the oXiris group, and 50 were included in the control group. Additionally, 52 (62.7%) patients were males, and 31 (37.3%) were females, with a median age of 69 years (IQR: 60.5–77.0 years).

The source of infection was pneumonia in 72 (86.7%) patients, urinary tract infection in 8 (9.6%), and other foci, including pancreatitis, infective endocarditis, and intra-abdominal infection, in 3 (3.6%).

Comorbidities included hypertension in 34.9% (*n* = 29), diabetes mellitus in 28.9% (*n* = 24), coronary artery disease in 27.7% (*n* = 23), chronic obstructive pulmonary disease in 10.8% (*n* = 9), chronic kidney disease in 37.3% (*n* = 31), malignancy in 41.0% (*n* = 34), and a history of cerebrovascular events in 19.3% (*n* = 16).

Baseline lactate levels, norepinephrine dose, SOFA-2 score, classic SOFA score, and mechanical ventilation rate were significantly higher in the oXiris group than in the control group. Table 1 shows between-group comparisons of the baseline variables.

Septic shock was present in 22/33 (66.7%) patients in the oXiris group and 28/50 (56.0%) patients in the control group, whereas sepsis was present in 11/33 (33.3%) and 22/50 (44.0%), respectively (Fisher’s exact *p* = 0.367). The distribution of infection sources was similar between the two groups. In the oXiris group, pneumonia was the predominant infection source (29/33, 87.9%), followed by urinary tract infection (2/33, 6.1%) and other infection sources (2/33, 6.1%). In the control group, pneumonia was present in 43/50 patients (86.0%), urinary tract infection in 6/50 (12.0%), and other infection sources in 1/50 (2.0%). The distribution of infection sources did not differ significantly between the groups (Fisher–Freeman–Halton exact test, *p* = 0.521). Although the oXiris group exhibited substantially greater acute physiological severity, including higher SOFA/SOFA-2 scores, greater norepinephrine requirements, higher lactate levels, and more frequent mechanical ventilation, these patients were also younger and had lower prevalences of hypertension and previous cerebrovascular disease than the control group. Thus, baseline differences were bidirectional rather than uniformly unfavorable to the oXiris group.

### 3.2. Temporal Changes in Clinical and Laboratory Parameters

**Figure 2:**
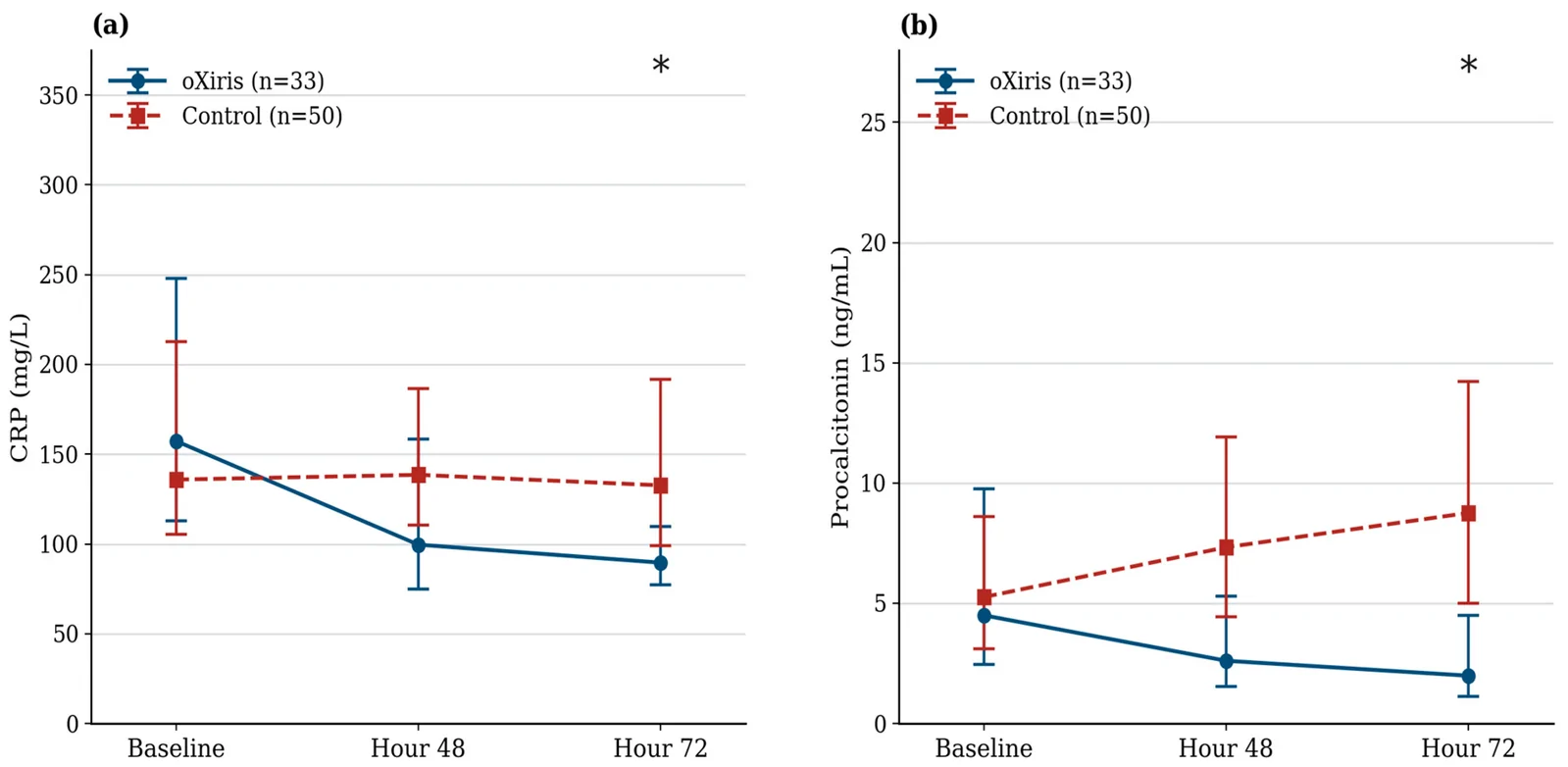
Temporal changes in inflammatory markers. (**a**) CRP levels. (**b**) PCT levels. Data points represent group medians, and error bars indicate 95% confidence intervals obtained by bootstrap resampling. * *p* < 0.05 indicates a statistically significant between-group difference.

Table 2 shows the clinical and laboratory parameters of the control and oXiris groups at baseline, 48 h, and 72 h. No significant difference in baseline CRP levels was observed between the two groups. The oXiris group showed a significant decrease in CRP levels over time, with the median CRP level decreasing from 157.30 mg/L at baseline to 99.60 mg/L at 48 h and 89.60 mg/L at 72 h. Conversely, no significant change in CRP levels was observed in the control group, and CRP levels did not show a marked reduction over time (Figure 2a).

The baseline PCT levels were comparable between the two groups. A significant decrease in PCT levels over time was observed in the oXiris group, with the median PCT level decreasing from 4.50 ng/mL at baseline to 1.99 ng/mL at 72 h. Conversely, no significant decline in PCT levels was observed in the control group (5.26 ng/mL at baseline and 8.76 ng/mL at 72 h) (Figure 2b).

**Figure 3:**
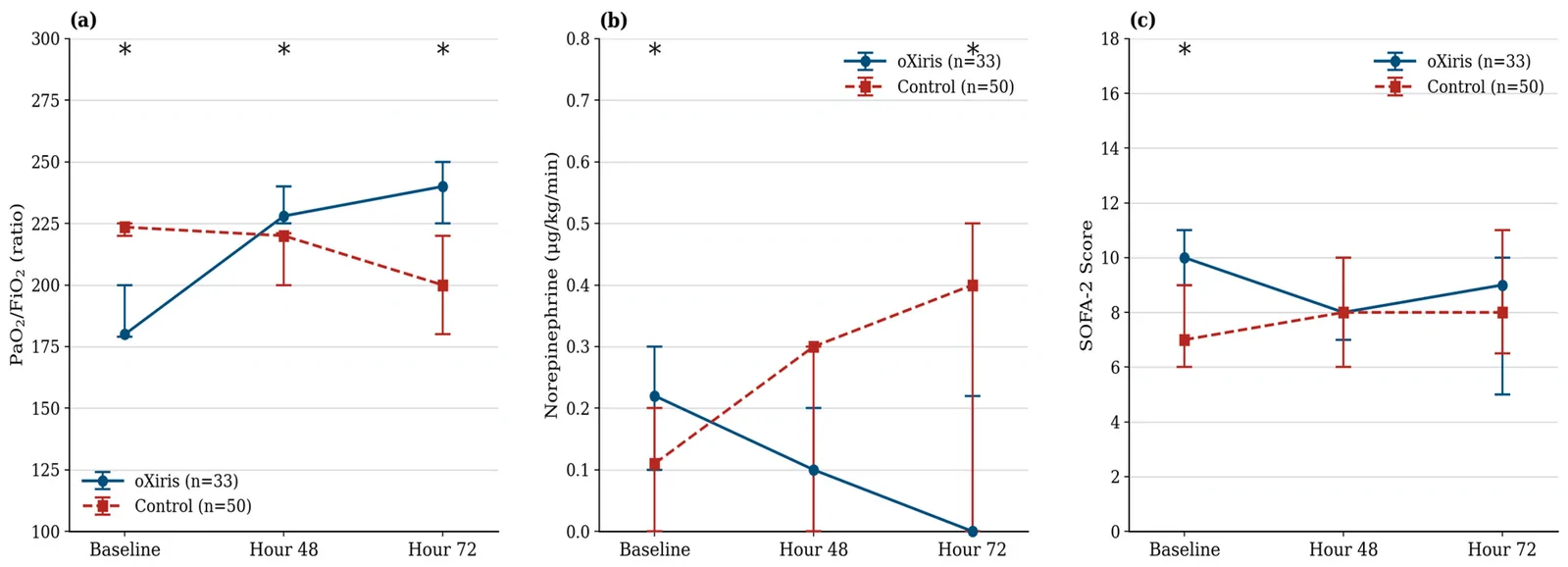
Temporal changes in clinical parameters. (**a**) PaO_2_/FiO_2_ ratio. (**b**) Norepinephrine dose. (**c**) SOFA-2 score. Data points represent group medians, and error bars indicate 95% confidence intervals obtained by bootstrap resampling. * *p* < 0.05 indicates a statistically significant between-group difference.

The baseline PaO_2_/FiO_2_ ratio was lower in the oXiris group than in the control group. However, a significant increase over time was observed in the oXiris group. The median PaO_2_/FiO_2_ ratio increased from 180 at baseline to 228 at 48 h and 240 at 72 h in the oXiris group, whereas it decreased from 223.5 at baseline to 200 at 72 h in the control group (Figure 3a).

Regarding hemodynamic support, the baseline norepinephrine dose was significantly higher in the oXiris group than in the control group. However, the norepinephrine dose decreased over time in the oXiris group and increased in the control group. The median norepinephrine dose decreased from 0.22 μg/kg/min at baseline to 0.00 μg/kg/min at 72 h in the oXiris group and increased from 0.11 μg/kg/min at baseline to 0.40 μg/kg/min at 72 h in the control group (Figure 3b).

The baseline SOFA-2 score was higher in the oXiris group than in the control group. The oXiris group showed a significant reduction in the SOFA-2 score over time, whereas the control group showed an increasing trend (Figure 3c). However, the absolute SOFA-2 score at 72 h did not differ significantly between the groups (median 9 [IQR 4.5–11] in the oXiris group vs. 8 [5,6,7,8,9,10,11,12] in the control group; *p* = 0.481).

**Figure 4:**
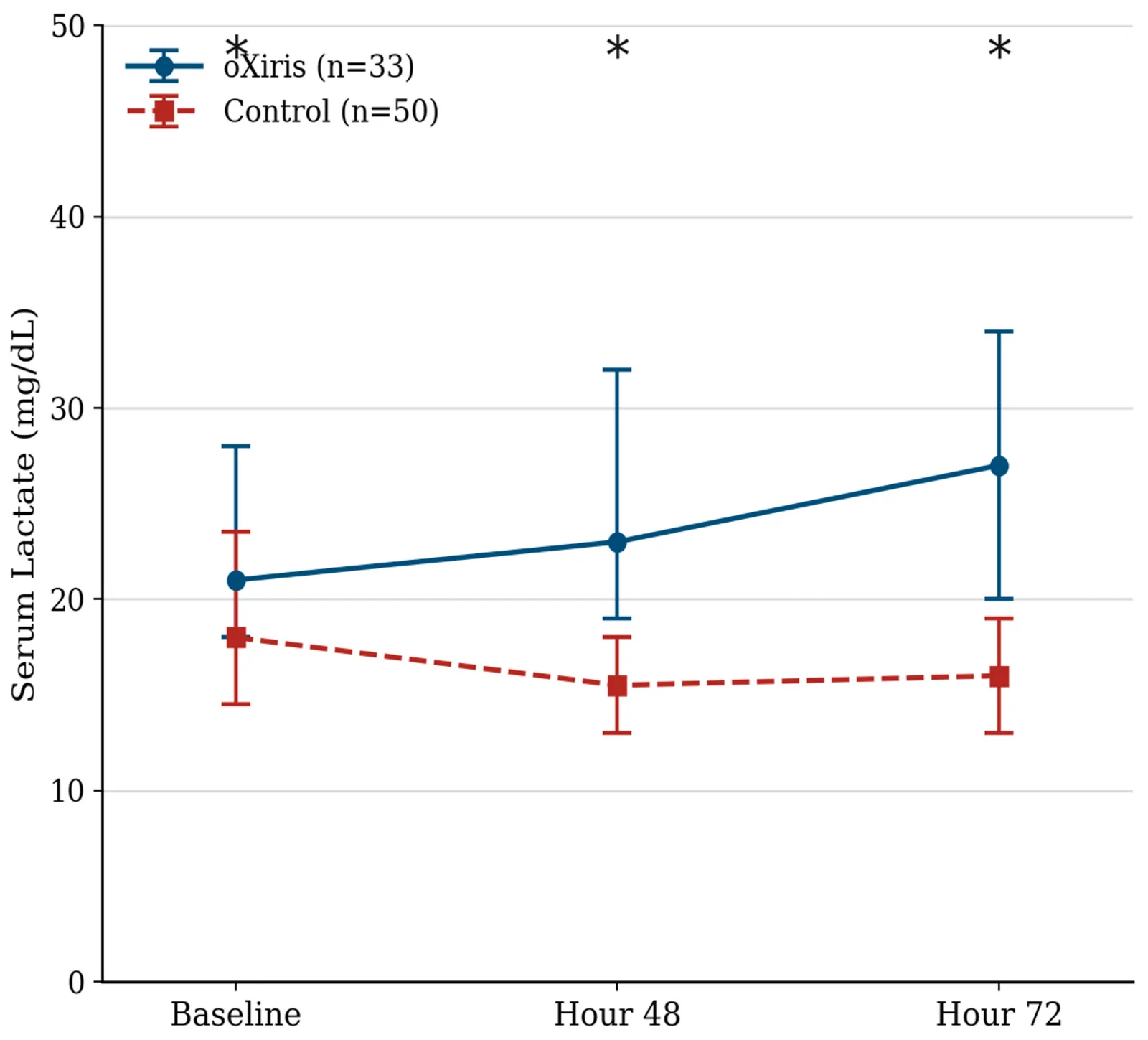
Temporal changes in serum lactate levels (mg/dL). Data points represent group medians, and error bars indicate 95% confidence intervals obtained by bootstrap resampling. * *p* < 0.05 indicates a statistically significant between-group difference.

Serum lactate levels were higher in the oXiris group than in the control group at all time points. Lactate showed an increasing trend in the oXiris group and a mild decline in the control group (Figure 4).

### 3.3. Group × Time Interaction Analyses

Adjusted GEE models were applied to assess whether temporal changes in clinical and laboratory parameters differed between groups. Table 3 shows overall group × time interaction *p*-values, Table 4 shows the adjusted regression coefficients (*β*) for these interactions, and Table 5 shows the adjusted between-group differences at each time point (48 and 72 h) with 95% CIs.

A significant group × time interaction was identified for the SOFA-2 score (Wald χ^2^ = 26.194, *p* < 0.001). The adjusted oXiris-control difference was −2.01 points (95% CI: −3.55 to −0.47; *p* = 0.010) at 48 h and −3.06 points (95% CI: −4.81 to −1.32; *p* < 0.001) at 72 h, indicating a significant difference in the temporal changes in the organ dysfunction score between the two groups.

A strong group × time interaction was observed for norepinephrine dose (Wald χ^2^ = 70.918, *p* < 0.001). The oXiris group had an adjusted norepinephrine dose 0.187 μg/kg/min (95% CI: 0.135–0.238; *p* < 0.001) lower at 48 h and 0.295 μg/kg/min (95% CI: 0.203–0.388; *p* < 0.001) lower at 72 h than the control group. Although the norepinephrine requirement decreased over time in the oXiris group, the control group showed an increasing trend.

A significant group × time interaction was identified for PaO_2_/FiO_2_ ratio (Wald χ^2^ = 101.707, *p* < 0.001). The oXiris group had an adjusted PaO_2_/FiO_2_ ratio 29.81 units (95% CI: 16.11–43.51; *p* < 0.001) higher at 48 h and 57.59 units (95% CI: 41.20–73.99; *p* < 0.001) higher at 72 h than the control group. Oxygenation improved markedly over time in the oXiris group, whereas the control group showed a declining trend.

Regarding inflammatory markers, significant group × time interactions were observed for CRP and PCT levels (Wald χ^2^ = 6.761, *p* = 0.034 and Wald χ^2^ = 7.989, *p* = 0.018, respectively). For CRP, the adjusted between-group difference at 48 h was not statistically significant (−41.06 mg/L; 95% CI: −87.02 to 4.91; *p* = 0.080), whereas the adjusted difference at 72 h was significant (−55.15 mg/L; 95% CI: −100.23 to −10.07; *p* = 0.016). The adjusted PCT differences were significant at both 48 h (−14.00 ng/mL; 95% CI: −23.61 to −4.39; *p* = 0.004) and 72 h (−13.24 ng/mL; 95% CI: −22.14 to −4.33; *p* = 0.004). The corresponding time-specific group × time coefficients (Table 4) were also significant for CRP at 48 h (*p* = 0.032) and 72 h (*p* = 0.043) and for PCT at 48 h (*p* = 0.003) and 72 h (*p* = 0.031); thus, for both inflammatory markers the two model outputs were directionally concordant.

A significant overall group × time interaction was identified for lactate levels (Wald χ^2^ = 6.344, *p* = 0.042). In contrast to the inflammatory markers, the two model outputs were discordant for lactate: the time-specific group × time coefficients (Table 4) did not reach statistical significance at 48 h (*p* = 0.118) or 72 h (*p* = 0.052), whereas the adjusted between-group differences (Table 5) were higher in the oXiris group at both 48 h (9.86 mg/dL; 95% CI: 2.74–16.97; *p* = 0.007) and 72 h (10.27 mg/dL; 95% CI: 3.26–17.28; *p* = 0.004). Because Table 4 estimates the between-group difference in change from baseline whereas Table 5 estimates the adjusted between-group difference in level at each time point, the latter remains influenced by the higher baseline lactate in the oXiris group. Under either specification, however, the observed lactate pattern was not in favor of oXiris. No significant group × time interaction was identified for WBC (Wald χ^2^ = 2.453, *p* = 0.293), and no adjusted between-group difference reached statistical significance at any time point.

Table 4 and Table 5 show the time-point-specific adjusted coefficients and group differences. *p*-values are interpreted as exploratory. No adjustment was applied for multiple comparisons.

### 3.4. Survival Analysis

**Figure 5:**
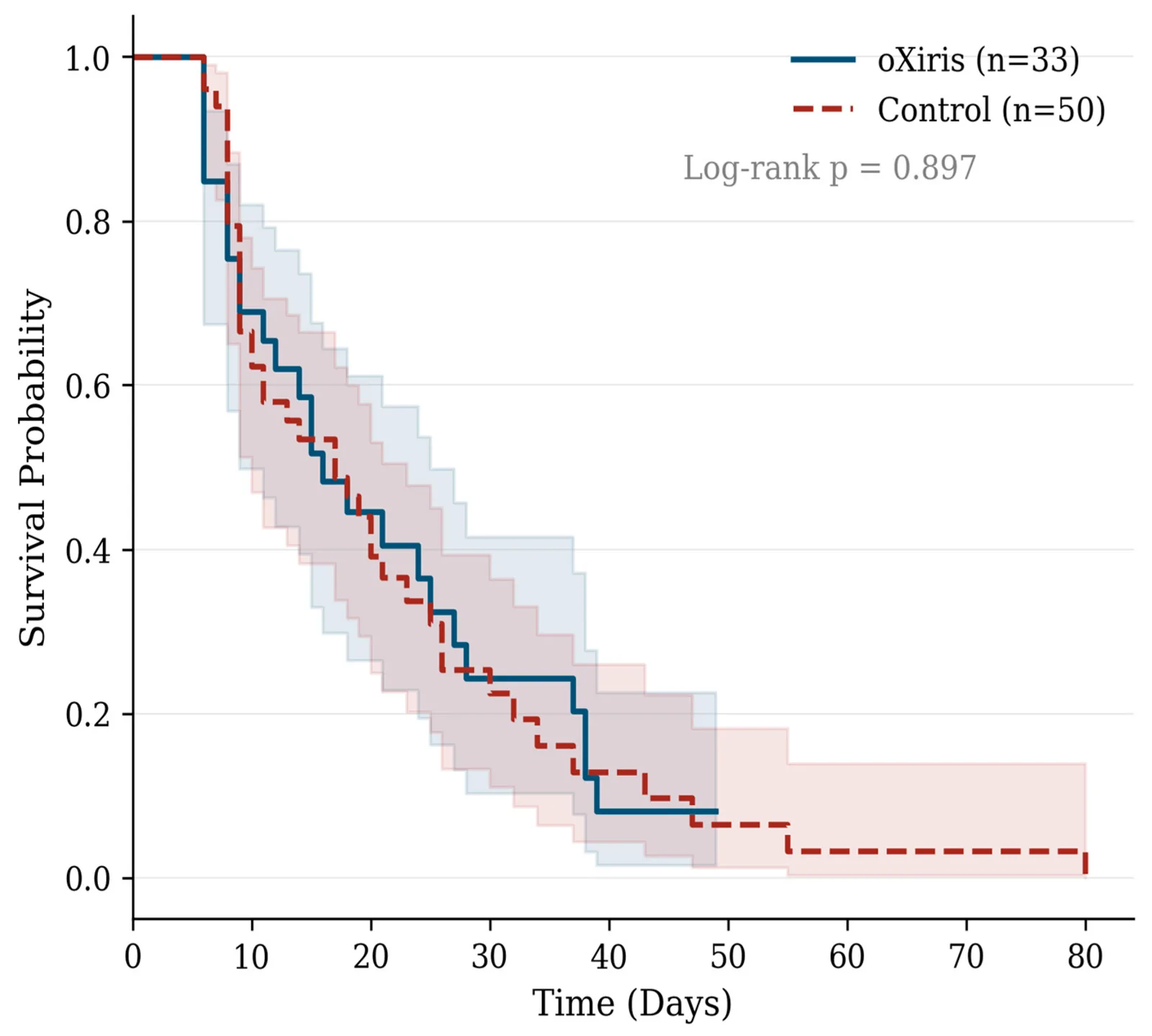
Kaplan–Meier survival curve (log-rank *p* = 0.897).

Kaplan–Meier survival analysis revealed no significant difference in OS between the oXiris and control groups (log-rank *p* = 0.897). The median OS was 17 days (95% CI: 9.6–24.4) in the control group and 16 days (95% CI: 10.9–21.1) in the oXiris group (Figure 5). Crude in-hospital mortality was 26/33 (78.8%) in the oXiris group and 41/50 (82.0%) in the control group (Fisher’s exact test, *p* = 0.780).

### 3.5. Renal Characteristics and Concomitant Renal Support 

At baseline, serum creatinine was elevated in both groups, with no significant between-group difference (oXiris 2.93 mg/dL [IQR 2.10–3.57] vs. control 2.10 mg/dL [1.31–3.54]; *p* = 0.157). A creatinine ≥ 2.0 mg/dL was present in 26/33 (78.8%) oXiris and 27/50 (54.0%) control patients (*p* = 0.039), indicating a higher baseline renal-dysfunction burden in the oXiris group, consistent with its greater overall severity. A history of chronic kidney disease was documented in 9/33 (27.3%) oXiris and 22/50 (44.0%) control patients (*p* = 0.190). Conventional intermittent hemodialysis for a renal indication was used in 3/33 (9.1%) oXiris and 15/50 (30.0%) control patients (*p* = 0.047); no control patient received oXiris or any other adsorptive membrane. Over the 72-h period, serum creatinine declined in the oXiris group (median 2.93 → 1.53 → 1.30 mg/dL) and was lower than in the control group at 48 h (1.53 vs. 2.07 mg/dL; *p* = 0.019) and 72 h (1.30 vs. 1.83 mg/dL; *p* = 0.029) (Table 6).

### 3.6. Propensity-Score Sensitivity Analysis

To address confounding by indication, a propensity score for oXiris use was estimated from baseline age, SOFA-2 score, lactate, norepinephrine dose, mechanical ventilation, and creatinine, and stabilized inverse-probability-of-treatment weights were applied. Weighting achieved good covariate balance, reducing all absolute standardized mean differences from up to 0.80 to ≤0.08. In IPTW-adjusted models, the oXiris-control differences in change from baseline to 72 h remained significant and directionally consistent for the SOFA-2 score (−3.52; 95% CI −4.53 to −2.51; *p* < 0.001), norepinephrine dose (−0.29 μg/kg/min; −0.38 to −0.19; *p* < 0.001), and PaO_2_/FiO_2_ ratio (+73.2; +54.2 to +92.1; *p* < 0.001). The differences in CRP (−43.6 mg/L; −99.1 to +11.8; *p* = 0.121) and PCT (−6.35 ng/mL; −14.29 to +1.59; *p* = 0.116) were attenuated and no longer reached significance, whereas the unfavorable lactate difference persisted (+6.65 mg/dL; +1.28 to +12.02; *p* = 0.016) (Table 7). Importantly, the IPTW analysis (Table 7) demonstrated that the improvements in SOFA-2 score, norepinephrine requirement, and PaO_2_/FiO_2_ ratio remained significant after adjustment, whereas the CRP and PCT differences were no longer statistically significant.

## 4. Discussion

This retrospective comparative cohort study evaluated the associations of adding oXiris hemoadsorption to standard sepsis therapy with early inflammatory, hemodynamic, respiratory, and organ dysfunction trajectories in patients with sepsis or septic shock in the ICU. The oXiris group showed more favorable temporal changes in SOFA-2 score, norepinephrine requirement, and PaO_2_/FiO_2_ ratio. However, the CRP and PCT differences were not maintained after IPTW adjustment, serum lactate showed an unfavorable pattern, and no significant difference in OS was observed. Given the observational design, these findings should not be interpreted as evidence of a causal treatment effect.

In sepsis and septic shock, circulating endotoxins trigger uncontrolled inflammatory responses by activating monocytes, macrophages, granulocytes, and endothelial cells, promoting cytokine release. This may result in complement and coagulation activation, microvascular dysfunction, tissue hypoxia, and multiple organ dysfunction syndrome [2]. Therefore, removing endotoxins and proinflammatory mediators from the circulation is a pathophysiologically rational therapeutic target. The principal extracorporeal blood purification modalities for this purpose include high-volume hemofiltration, plasma exchange, immunoadsorption, and adsorptive membrane technologies [7,8].

The oXiris membrane provides cytokine adsorption through its modified AN69 hydrogel structure and endotoxin-binding capacity via its positively charged polyethyleneimine coating. This dual adsorptive property may reduce the inflammatory mediator burden in sepsis, contributing to hemodynamic stabilization and organ function improvement [9,19]. However, numerous variables influence the effect of adsorptive therapies on clinical outcomes, including patient selection, treatment timing, infection source, source control, antimicrobial appropriateness, organ support requirements, and disease severity, because sepsis is a complex and heterogeneous clinical syndrome.

Current literature and clinical consensus guidelines recommend evaluating early response to oXiris therapy through vasopressor requirements, PaO_2_/FiO_2_ ratio, lactate levels, inflammatory markers, and organ dysfunction scores [20,21]. In their study comparing conventional CRRT with oXiris membrane-based therapy, Zhai et al. reported marked reductions from baseline in heart rate, respiratory rate, norepinephrine dose, lactate, PCT, and SOFA score on the third day of treatment in the oXiris group [22]. Similarly, several clinical studies have shown that oXiris therapy is associated with improvements in hemodynamic parameters, reduction in norepinephrine requirement, enhanced lactate clearance, decreased fluid requirements, and lower SOFA scores [23,24,25,26,27].

Notably, the oXiris group presented with greater baseline clinical severity than the control group. The baseline lactate level, norepinephrine dose, SOFA-2 score, classic SOFA score, and mechanical ventilation rate were significantly higher in the oXiris group than in the control group. This finding indicates that oXiris therapy is preferred in real-world practice for patients with more severe clinical presentations, higher vasopressor requirements, and more pronounced organ dysfunction. Therefore, baseline differences in disease severity should be considered when interpreting the findings of this study.

Importantly, the baseline imbalance should be interpreted in both directions. Although patients treated with oXiris had substantially greater acute physiological severity, they were also younger and had lower prevalences of hypertension and previous cerebrovascular disease. These opposing baseline characteristics should be considered when interpreting the observed associations, and residual confounding cannot be completely excluded despite adjusted and IPTW analyses.

A central point requiring clarification concerns the indication for and mode of oXiris use in this cohort. Although oXiris is most often described as a renal-replacement membrane for septic AKI, in our practice it was applied as adjunctive hemoadsorption driven by a hyperinflammatory, vasopressor-dependent phenotype rather than by a renal indication; the CVVHDF circuit served only as the delivery platform for endotoxin and cytokine adsorption. Consistent with real-world use, the oXiris group carried a heavier baseline burden of renal dysfunction (creatinine ≥2.0 mg/dL in 78.8% vs. 54.0%), yet the comparator arm received standard therapy without any adsorptive membrane rather than an alternative purification filter. Although serum creatinine declined over time in the oXiris group, this finding should not be interpreted as evidence of renal recovery alone. Because oXiris therapy was delivered through a CVVHDF circuit, part of the observed reduction in serum creatinine may simply reflect extracorporeal solute clearance rather than true restoration of intrinsic kidney function. Renal parameters were included as secondary laboratory outcomes rather than to evaluate renal recovery. Therefore, the observed changes in serum creatinine should not be interpreted as evidence of improved kidney function. Because KDIGO staging and urine output data were outside the scope of the present study, no conclusions regarding renal recovery can be drawn. While this trajectory is compatible with attenuation of the septic insult, it should be interpreted cautiously given the non-randomized design, the baseline imbalance, and the descriptive nature of the renal data.

An important observation of the present study was that reductions in CRP and PCT were no longer statistically significant after IPTW adjustment, whereas improvements in SOFA-2 score, norepinephrine requirement, and PaO_2_/FiO_2_ ratio remained significant. This suggests that the latter findings were more robust to adjustment for baseline differences and may therefore have greater clinical relevance. Conversely, the inflammatory marker findings appear more susceptible to residual baseline imbalance and should be interpreted cautiously. The unexpected increase in lactate levels also warrants cautious interpretation and may have been influenced by factors beyond disease severity, including exposure to lactate-containing CRRT solutions.

Regarding inflammatory markers, CRP and PCT levels decreased markedly over time in the oXiris group in the unweighted analyses. However, the attenuation of CRP and PCT differences after IPTW suggests that these findings were more susceptible to baseline imbalances than the observed changes in SOFA-2 score, norepinephrine requirement, and oxygenation. This may indicate that the apparent reductions in inflammatory biomarkers were influenced, at least in part, by differences in baseline clinical characteristics rather than reflecting an independent treatment effect. Reductions in CRP and procalcitonin should also be interpreted cautiously because adsorption and extracorporeal clearance through the oXiris membrane may contribute to lower circulating concentrations independently of true resolution of systemic inflammation.

Previous clinical studies examining oXiris or similar extracorporeal hemoadsorption therapies have reported improvements in conventional SOFA scores [15]. In our study, a significant group × time interaction was observed for the SOFA-2 score. However, the absolute SOFA-2 score at 72 h was not significantly different between the groups (median 9 [IQR 4.5–11] in the oXiris group vs. 8 [5,6,7,8,9,10,11,12] in the control group; *p* = 0.481). Because the oXiris group started with a higher SOFA-2 score, the greater observed decline may partly reflect regression to the mean. The result should therefore be interpreted as a difference in temporal trajectory rather than an absolute between-group advantage at 72 h. Although the conventional SOFA has long been used to assess organ dysfunction in sepsis, the SOFA-2 was used in this study because it reflects contemporary ICU practice [17].

Regarding respiratory parameters, the baseline PaO_2_/FiO_2_ ratio was lower in the oXiris group but improved markedly at 48 and 72 h. The PaO_2_/FiO_2_ ratio increased from 180 to 240 in the oXiris group and declined from 223.5 to 200 in the control group. This finding indicates that oXiris therapy is associated with early improvements in oxygenation parameters. Cytokine-mediated alveolar inflammation, pulmonary endothelial injury, and increased capillary permeability play important roles in sepsis-associated acute lung injury. The endotoxin and cytokine adsorption capacity of the oXiris membrane may help reduce the pulmonary inflammatory burden. The Asia-Pacific oXiris Expert Meeting 2024 consensus statements identified early improvement in PaO_2_/FiO_2_ ratio as one of the key parameters for monitoring treatment response [20].

From a hemodynamic perspective, the temporal change in norepinephrine requirements was one of the most striking findings of our study. Despite higher baseline norepinephrine doses in the oXiris group, the median norepinephrine dose declined to 0.00 μg/kg/min by 72 h. Conversely, norepinephrine requirements increased over time in the control group. The identification of a strong group × time interaction for norepinephrine dose in the GEE analyses demonstrates that the trajectory of change in hemodynamic support requirements differed markedly between the two groups. These findings are consistent with those of previous studies, which showed that oXiris therapy is associated with reduced vasopressor requirements in septic shock [13,23,28,29].

The serum lactate results were the most important finding diverging from the literature. Although lactate clearance improvement might be expected after successful resuscitation and reduction in inflammatory mediator burden, lactate levels showed an increasing trend in the oXiris group. Lactate levels increased from 21 mg/dL at baseline to 27 mg/dL at 72 h in the oXiris group and decreased from 18 mg/dL to 16 mg/dL in the control group. GEE analysis identified a significant group × time interaction for lactate. However, this change was not in favor of oXiris.

This finding may be attributed to several mechanisms, but these explanations remain hypothetical. The higher baseline SOFA-2 score, norepinephrine requirement, and mechanical ventilation rate in the oXiris group indicate greater baseline physiological severity. Even when macrohemodynamic parameters improve in septic shock, microcirculatory and cellular metabolic recovery may occur later. Castro et al. reported that lactate kinetics may follow a biphasic course and may reflect adrenergic stimulation, mitochondrial dysfunction, and hepatic clearance in addition to tissue hypoperfusion [30]. Alternative explanations, including patient selection, baseline illness severity, hepatic lactate clearance, differences in fluid resuscitation and ultrafiltration, and other unmeasured aspects of critical care management, may also have contributed to the observed lactate pattern. Another potential contributor to the unexpected lactate trajectory is the composition of the CRRT fluids used during oXiris-CVVHDF. The dialysis solutions used across the cohort included Prism0cal B22, Prism0cal, and Dialisan, all of which contained 3 mmol/L lactate after reconstitution. Therefore, exposure to lactate-containing CRRT fluids may have influenced serum lactate kinetics and may have contributed to the absence of the expected lactate decline. However, the magnitude and direction of this contribution could not be quantified because solution-specific exposure and cumulative delivered volumes were not included as patient-level variables in the retrospective analysis. Accordingly, the observed lactate trajectory should not be attributed solely to disease severity or to the biological effect of oXiris and should be interpreted cautiously.

Evidence regarding the impact of oXiris therapy on mortality is heterogeneous. Some retrospective studies have shown that oXiris therapy is associated with a reduction in 28-day mortality [28]. A meta-analysis by Wang et al. that evaluated 14 studies and 695 patients showed that oXiris use was associated with lower 28-day mortality. However, the authors emphasized that the low quality of available evidence warrants confirmation in larger-scale studies [15]. In this study, no significant difference in OS was observed between the control and oXiris groups (median survival: 17 vs. 16 days). This finding indicates that, despite early favorable temporal changes in inflammatory, hemodynamic, and respiratory parameters with oXiris therapy, these early differences were not accompanied by a difference in survival. This outcome may be attributed to the greater baseline disease severity in the oXiris group, the limited sample size, and the retrospective study design. Because only patients who survived the first 72 h and remained evaluable during this period were included, shifting the time origin from diagnosis to 72 h would change the time axis equally for all patients and would not alter the relative survival comparison between groups. Therefore, the original Kaplan–Meier analysis was retained, while the event definition, censoring method, and crude mortality by group were explicitly reported. The low survival probabilities shown in Figure 5 therefore reflect the genuinely high in-hospital mortality observed in both groups (78.8% and 82.0%) rather than the misclassification of discharged patients as deaths; all patients discharged alive were censored at the date of discharge.

This study has several limitations. Treatment selection was based on clinician assessment rather than predefined standardized objective criteria, creating a risk of confounding by indication and selection bias. Although adjusted GEE and IPTW analyses were applied to balance measured baseline differences, several clinically relevant variables, including antimicrobial appropriateness, adequacy of infection-source control, cumulative fluid balance, corticosteroid use, and mechanical ventilation strategies, were not systematically recorded in the retrospective dataset and therefore could not be included in the analyses. Residual confounding therefore cannot be excluded. In addition, because the primary objective was to evaluate treatment-associated temporal changes over a predefined 72-h period, inclusion required survival and evaluability through this assessment window. Consequently, patients who died before 72 h or did not complete the predefined treatment and assessment period were excluded (Figure 1). We explicitly acknowledge that this requirement may have introduced survivor/selection bias by preferentially retaining patients who survived and remained evaluable through 72 h. Accordingly, the primary longitudinal findings should be interpreted as 72-h trajectories among patients who completed this assessment window, and caution is warranted when extrapolating these findings to patients with very early clinical deterioration or death. Because these patients were by definition not eligible for the protocol-specified 48- and 72-h serial assessments, the repeated measurements required for a formal sensitivity analysis including them do not exist, and any such analysis would have relied on imputation under untestable assumptions; the point at which these patients left the eligibility pathway is therefore made explicit in the patient flow diagram (Figure 1) instead.

The single-center design and limited sample size may also reduce generalizability and statistical power, particularly for survival outcomes. In addition, complete organism-level microbiological data were not systematically available in the retrospective dataset. Therefore, subgroup analyses according to these variables could not be performed and should be addressed in future prospective studies. Although Prism0cal B22, Prism0cal, and Dialisan were used as CRRT solutions during oXiris-CVVHDF, solution-specific exposure and cumulative delivered volumes were not available as patient-level analytical variables. Therefore, the quantitative contribution of their lactate content to serum lactate kinetics could not be determined retrospectively.

Given the retrospective observational design and the potential for residual confounding, our findings should be considered hypothesis-generating rather than evidence of a causal treatment effect. Confirmation in prospective randomized studies is warranted.

## 5. Conclusions

In this retrospective cohort, the addition of oXiris hemoadsorption to standard sepsis therapy was associated with favorable early temporal changes in SOFA-2 score, norepinephrine requirement, and PaO_2_/FiO_2_ ratio. However, the observed differences in CRP and PCT were not maintained after IPTW adjustment, and no survival benefit was demonstrated. Given the observational design, nonrandom treatment allocation, and potential for residual confounding, these findings should be considered hypothesis-generating and should not be interpreted as evidence of a causal treatment effect. Prospective randomized studies are required to confirm these associations.

## Figures and Tables

**Table 1:** Between-group comparison of the baseline variables.

Baseline Variables	Control Group (*n* = 50)	oXiris Group (*n* = 33)	*p*-Value
Age, years, median (IQR)	72 (64.75–79)	65 (56–72)	0.005
Female sex, *n* (%)	19 (38.0)	12 (36.4)	1.000
Male sex, *n* (%)	31 (62.0)	21 (63.6)	1.000
Hypertension, *n* (%)	22 (44.0)	7 (21.2)	0.037
Diabetes mellitus, *n* (%)	17 (34.0)	7 (21.2)	0.229
Coronary artery disease, *n* (%)	16 (32.0)	7 (21.2)	0.325
Chronic obstructive pulmonary disease, *n* (%)	7 (14.0)	2 (6.1)	0.306
Chronic kidney disease, *n* (%)	22 (44.0)	9 (27.3)	0.165
Cerebrovascular event, *n* (%)	14 (28.0)	2 (6.1)	0.021
Malignancy, *n* (%)	20 (40.0)	14 (42.4)	1.000
Septic shock, *n* (%)	28 (56.0)	22 (66.7)	0.367
Sepsis, *n* (%)	22 (44.0)	11 (33.3)	—
Infection source: pneumonia, *n* (%)	43 (86.0)	29 (87.9)	0.521
Infection source: urinary tract infection, *n* (%)	6 (12.0)	2 (6.1)	—
Infection source: other, *n* (%)	1 (2.0)	2 (6.1)	—
Baseline SOFA-2 score, median (IQR)	7 (5–10)	10 (7.5–13)	0.004
Classic SOFA score, median (IQR)	6 (4–8)	9 (5–11)	0.009
Mechanical ventilation, *n* (%)	16 (32.0)	23 (69.7)	0.001
Baseline lactate level, mg/dL, median (IQR)	18 (12–27)	21 (15.5–37)	0.045
Baseline norepinephrine dose, μg/kg/min, median (IQR)	0.11 (0.00–0.20)	0.22 (0.00–0.40)	0.017
Creatinine, mg/dL, median (IQR)	2.10 (1.31–3.54)	2.93 (2.10–3.57)	0.157

Continuous variables were compared using the Mann–Whitney U test, whereas categorical variables were compared using Fisher’s exact test. The distribution of infection sources was compared using the Fisher–Freeman–Halton exact test. IQR, interquartile range; SOFA, Sequential Organ Failure Assessment; SOFA-2, updated Sequential Organ Failure Assessment.

**Table 2:** Temporal and between-group changes in clinical and laboratory parameters.

Parameter/Time	Control Group (*n* = 50) Median (IQR)	oXiris Group (*n* = 33) Median (IQR)	Between-Group *p*
SOFA-2 score			
Baseline	7.00 (5.00–10.00) ^a^	10.00 (7.50–13.00) ^a^	0.004
48 h	8.00 (4.00–11.00) ^a^	8.00 (5.00–11.00) ^b^	0.801
72 h	8.00 (5.00–12.00) ^b^	9.00 (4.50–11.00) ^b^	0.481
Within-group *p*	0.001	<0.001	
PaO_2_/FiO_2_			
Baseline	223.50 (207.50–230.00) ^a^	180.00 (170.00–200.00) ^a^	<0.001
48 h	220.00 (195.00–226.50) ^b^	228.00 (222.50–245.00) ^b^	0.001
72 h	200.00 (150.00–226.25) ^c^	240.00 (225.00–275.00) ^c^	<0.001
Within-group *p*	0.008	<0.001	
Norepinephrine dose, μg/kg/min			
Baseline	0.11 (0.00–0.20) ^a^	0.22 (0.00–0.40) ^a^	0.017
48 h	0.30 (0.00–0.40) ^b^	0.10 (0.00–0.25) ^b^	0.175
72 h	0.40 (0.00–0.60) ^c^	0.00 (0.00–0.40) ^ab^	0.019
Within-group *p*	<0.001	0.001	
Lactate, mg/dL			
Baseline	18.00 (12.00–27.00)	21.00 (15.50–37.00)	0.045
48 h	15.50 (10.00–21.00)	23.00 (16.00–40.00)	<0.001
72 h	16.00 (11.00–23.50)	27.00 (17.00–46.00)	0.001
Within-group *p*	0.177	0.077	
WBC, cells/μL			
Baseline	10,835 (7345–17,770)	18,310 (10,570–25,510)	0.011
48 h	11,405 (8187–18,340)	15,420 (7805–22,535)	0.190
72 h	10,410 (7135–14,517)	14,480 (8425–22,415)	0.073
Within-group *p*	0.922	0.079	
CRP, mg/L			
Baseline	135.85 (75.32–291.85)	157.30 (84.30–280.31) ^a^	0.552
48 h	138.50 (84.32–213.90)	99.60 (59.90–191.73) ^b^	0.142
72 h	132.65 (89.75–206.47)	89.60 (56.50–143.70) ^b^	0.012
Within-group *p*	0.684	<0.001	
Procalcitonin, ng/mL			
Baseline	5.26 (2.17–13.37)	4.50 (1.57–17.71) ^a^	0.845
48 h	7.33 (1.60–16.00)	2.61 (1.03–10.49) ^b^	0.073
72 h	8.76 (2.35–16.45)	1.99 (0.70–12.43) ^b^	0.012
Within-group *p*	0.298	<0.001	

Values are presented as median (IQR). Between-group comparisons were performed using the Mann–Whitney U test. Within-group temporal changes were evaluated using the Friedman test. Values in the same column sharing different superscripts (a–c) indicate statistically significant differences between time points (*p* < 0.05, Bonferroni-corrected Wilcoxon signed-rank test). CRP: C-reactive protein; WBC: white blood cell; PaO_2_/FiO_2_: ratio of arterial partial pressure of oxygen to the fraction of inspired oxygen. IQR, interquartile range; SOFA-2, updated Sequential Organ Failure Assessment.

**Table 3:** Temporal changes in clinical and laboratory parameters and adjusted GEE group × time interaction analysis.

Variable	Group	Baseline Median (IQR)	48 h Median (IQR)	72 h Median (IQR)	Adjusted GEE Group × Time *p*
SOFA-2 score	oXiris (*n* = 33)	10 (7.5–13)	8 (5–11)	9 (4.5–11)	<0.001
	Control (*n* = 50)	7 (5–10)	8 (4–11)	8 (5–12)	
Lactate, mg/dL	oXiris (*n* = 33)	21 (15.5–37)	23 (16–40)	27 (17–46)	0.042
	Control (*n* = 50)	18 (12–27)	15.5 (10–21)	16 (11–23.5)	
Norepinephrine, μg/kg/min	oXiris (*n* = 33)	0.22 (0.00–0.40)	0.10 (0.00–0.25)	0.00 (0.00–0.40)	<0.001
	Control (*n* = 50)	0.11 (0.00–0.20)	0.30 (0.00–0.40)	0.40 (0.00–0.60)	
PaO_2_/FiO_2_	oXiris (*n* = 33)	180 (170–200)	228 (222.5–245)	240 (225–275)	<0.001
	Control (*n* = 50)	223.5 (207.5–230)	220 (195–226.5)	200 (150–226.25)	
WBC, cells/μL	oXiris (*n* = 33)	18,310 (10,570–25,510)	15,420 (7805–22,535)	14,480 (8425–22,415)	0.293
	Control (*n* = 50)	10,835 (7345–17,770)	11,405 (8187–18,340)	10,410 (7135–14,517)	
CRP, mg/L	oXiris (*n* = 33)	157.30 (84.30–280.31)	99.60 (59.90–191.73)	89.60 (56.50–143.70)	0.034
	Control (*n* = 50)	135.85 (75.32–291.85)	138.50 (84.32–213.90)	132.65 (89.75–206.47)	
Procalcitonin, ng/mL	oXiris (*n* = 33)	4.50 (1.57–17.71)	2.61 (1.03–10.49)	1.99 (0.70–12.43)	0.018
	Control (*n* = 50)	5.26 (2.17–13.37)	7.33 (1.60–16.00)	8.76 (2.35–16.45)	

Data are presented as median (IQR). The adjusted group × time *p*-value reflects the *p*-value for the group × time interaction term in the GEE model. To prevent overadjustment and multicollinearity, covariates for inflammatory and respiratory parameters were age, mechanical ventilation status, baseline SOFA-2 score, and lactate level, and covariates for hemodynamic and organ dysfunction parameters were age, mechanical ventilation status, lactate/SOFA-2 score (depending on the dependent variable), and baseline norepinephrine dose. GEE: generalized estimating equations; CRP: C-reactive protein; WBC: white blood cell; PaO_2_/FiO_2_: ratio of arterial partial pressure of oxygen to the fraction of inspired oxygen. IQR, interquartile range; SOFA-2, updated Sequential Organ Failure Assessment.

**Table 4:** Adjusted GEE group × time interaction coefficients (adjusted *β*, 95% CI) for primary outcome parameters.

Variable	Unit	48 h: Group × Time *β* (95% CI; *p*)	72 h: Group × Time *β* (95% CI; *p*)
SOFA-2 score	points	−2.10 (−2.89 to −1.31; *p* < 0.001)	−3.15 (−4.22 to −2.08; *p* < 0.001)
Lactate	mg/dL	6.3 (−1.6 to 14.2; *p* = 0.118)	6.7 (−0.1 to 13.4; *p* = 0.052)
Norepinephrine	μg/kg/min	−0.176 (−0.225 to −0.127; *p* < 0.001)	−0.284 (−0.376 to −0.192; *p* < 0.001)
PaO_2_/FiO_2_	ratio	50.6 (40.1 to 61.1; *p* < 0.001)	78.4 (63.0 to 93.8; *p* < 0.001)
WBC	10^3^/μL	−2.22 (−5.25 to 0.80; *p* = 0.149)	−2.06 (−6.15 to 2.03; *p* = 0.323)
CRP	mg/L	−46.22 (−88.41 to −4.03; *p* = 0.032)	−60.31 (−118.61 to −2.01; *p* = 0.043)
Procalcitonin	ng/mL	−10.09 (−16.79 to −3.38; *p* = 0.003)	−9.32 (−17.80 to −0.84; *p* = 0.031)

*β* coefficients indicate how much the change from baseline at the given time point differs between the oXiris and control groups. Negative values indicate a greater reduction in the oXiris group, whereas positive values indicate a greater increase. All models were adjusted with the following covariates: age, mechanical ventilation status, baseline SOFA-2 score, and lactate level were covariates for inflammatory and respiratory parameters, and age, mechanical ventilation status, lactate/SOFA-2 score (depending on the dependent variable), and baseline norepinephrine dose were covariates for hemodynamic and organ dysfunction parameters. Lactate levels are reported in mg/dL as available in the institutional laboratory information system. GEE: generalized estimating equations; CI: confidence interval; CRP: C-reactive protein; WBC: white blood cell; procalcitonin. SOFA-2, updated Sequential Organ Failure Assessment.

**Table 5:** Adjusted oXiris-control group differences at 48 and 72 h derived from adjusted GEE models (with 95% CI).

Variable	Unit	48 h: Adjusted Difference (95% CI; *p*)	72 h: Adjusted Difference (95% CI; *p*)
SOFA-2 score	points	−2.01 (−3.55 to −0.47; *p* = 0.010)	−3.06 (−4.81 to −1.32; *p* < 0.001)
Lactate	mg/dL	9.86 (2.74 to 16.97; *p* = 0.007)	10.27 (3.26 to 17.28; *p* = 0.004)
Norepinephrine	μg/kg/min	−0.187 (−0.238 to −0.135; *p* < 0.001)	−0.295 (−0.388 to −0.203; *p* < 0.001)
PaO_2_/FiO_2_	ratio	29.81 (16.11 to 43.51; *p* < 0.001)	57.59 (41.20 to 73.99; *p* < 0.001)
WBC	10^3^/μL	3.10 (−1.99 to 8.19; *p* = 0.232)	3.27 (−2.36 to 8.89; *p* = 0.255)
CRP	mg/L	−41.06 (−87.02 to 4.91; *p* = 0.080)	−55.15 (−100.23 to −10.07; *p* = 0.016)
Procalcitonin	ng/mL	−14.00 (−23.61 to −4.39; *p* = 0.004)	−13.24 (−22.14 to −4.33; *p* = 0.004)

Differences are presented in the oXiris-control direction. Negative differences in the SOFA-2 score, norepinephrine, CRP level, and PCT level and a positive difference in PaO_2_/FiO_2_ are interpreted as favorable for the oXiris group. A positive difference in lactate level indicates higher lactate levels in the oXiris group and is not interpreted as treatment-favorable. *p*-values are exploratory. No adjustment was applied for multiple comparisons. CI: confidence interval; CRP: C-reactive protein; WBC: white blood cell; SOFA-2, updated Sequential Organ Failure Assessment.

**Table 6:** Renal characteristics and concomitant renal support use by group.

Renal Parameter	Control Group (*n* = 50)	oXiris Group (*n* = 33)	*p*-Value
Baseline creatinine, mg/dL, median (IQR)	2.10 (1.31–3.54)	2.93 (2.10–3.57)	0.157
Creatinine ≥ 2.0 mg/dL, *n* (%)	27 (54.0)	26 (78.8)	0.035
Chronic kidney disease, n (%)	22 (44.0)	9 (27.3)	0.165
Conventional intermittent hemodialysis, *n* (%)	15 (30.0)	3 (9.1)	0.030
Creatinine at 48 h, mg/dL, median (IQR)	2.07 (1.31–3.47)	1.53 (0.79–1.99)	0.019
Creatinine at 72 h, mg/dL, median (IQR)	1.83 (1.19–3.29)	1.30 (0.90–1.90)	0.029

Between-group comparisons used the Mann–Whitney U test (continuous variables) or the chi-square test (categorical variables). Conventional intermittent hemodialysis denotes dialysis provided for a renal indication; no control patient received oXiris or any other adsorptive membrane. Formal KDIGO AKI staging was not feasible owing to the absence of reliable pre-morbid baseline creatinine. IQR, interquartile range.

**Table 7:** Inverse-probability-of-treatment-weighted (IPTW) sensitivity analysis of the primary outcomes.

Parameter (Δ Baseline → 72 h)	IPTW-Adjusted oXiris-Control Difference (95% CI)	*p*-Value
SOFA-2 score, points	−3.52 (−4.53 to −2.51)	<0.001
Norepinephrine, μg/kg/min	−0.29 (−0.38 to −0.19)	<0.001
PaO_2_/FiO_2_ ratio	+73.2 (+54.2 to +92.1)	<0.001
CRP, mg/L	−43.6 (−99.1 to +11.8)	0.121
Procalcitonin, ng/mL	−6.35 (−14.29 to +1.59)	0.116
Lactate, mg/dL	+6.65 (+1.28 to +12.02)	0.016

Differences are oXiris-control in change from baseline to 72 h, estimated by weighted least squares with stabilized inverse-probability-of-treatment weights. The propensity score included baseline age, SOFA-2 score, lactate, norepinephrine dose, mechanical ventilation, and creatinine, and reduced all absolute standardized mean differences from up to 0.80 to ≤0.08. Negative values for SOFA-2, norepinephrine, CRP, and procalcitonin and positive values for PaO_2_/FiO_2_ favor oXiris; a positive lactate difference is not treatment-favorable. SOFA-2, updated Sequential Organ Failure Assessment; CI, confidence interval; IPTW, inverse-probability-of-treatment-weighting; CRP, C-reactive protein.

## Data Availability

The data used in this study cannot be publicly shared due to ethical and institutional restrictions. Data may be obtained from the corresponding author upon reasonable request, subject to applicable ethics committee and institutional approvals.
